# Predictors of Patient-Reported Incontinence at Adjuvant/Salvage Radiotherapy after Prostatectomy: Impact of Time between Surgery and Radiotherapy

**DOI:** 10.3390/cancers13133243

**Published:** 2021-06-29

**Authors:** Fernando Munoz, Giuseppe Sanguineti, Andrea Bresolin, Domenico Cante, Vittorio Vavassori, Justina Magdalena Waskiewicz, Giuseppe Girelli, Barbara Avuzzi, Elisabetta Garibaldi, Adriana Faiella, Elisa Villa, Alessandro Magli, Barbara Noris Chiorda, Marco Gatti, Tiziana Rancati, Riccardo Valdagni, Nadia G. Di Muzio, Claudio Fiorino, Cesare Cozzarini

**Affiliations:** 1 SC Radioterapia Oncologica, Ospedale Regionale Parini-AUSL Valle d’Aosta, 11100 Aosta, Italy; fmunoz@ausl.vda.it; 2 Deptartment of Radiotherapy, IRCCS Istituto Nazionale dei Tumori “Regina Elena”, 00144 Roma, Italy; giuseppe.sanguineti@ifo.gov.it (G.S.); adriana.faiella@ifo.gov.it (A.F.); 3 Deptartment of Medical Physics and Deptartment of Radiotherapy, IRCCS Istituto Scientifico Ospedale San Raffaele, 20132 Milano, Italy; breso88@gmail.com (A.B.); dimuzio.nadia@hsr.it (N.G.D.M.); cozzarini.cesare@hsr.it (C.C.); 4 Deptartment of Radiotherapy, ASL TO4, Ospedale di Ivrea, 10015 Ivrea, Italy; dcante@aslto4.piemonte.it; 5 Deptartment of Radiotherapy, Cliniche Gavazzeni-Humanitas, 24125 Bergamo, Italy; vittorio.vavassori@gavazzeni.it (V.V.); elisa.villa@gavazzeni.it (E.V.); 6 Deptartment of Radiotherapy, Azienda Sanitaria dell’Alto Adige, 39100 Bolzano, Italy; justynamagdalena.waskiewicz@sabes.it; 7 Deptartment of Radiotherapy, Ospedale degli Infermi, 22399 Biella, Italy; Giuseppe.Girelli@aslbi.piemonte.it; 8 Deptartment of Radiation Oncology 1, Fondazione IRCCS Istituto Nazionale dei Tumori, 20133 Milan, Italy; Barbara.Avuzzi@istitutotumori.mi.it (B.A.); Barbara.Noris@istitutotumori.mi.it (B.N.C.); Tiziana.Rancati@istitutotumori.mi.it (T.R.); Riccardo.valdagni@istitutotumori.mi.it (R.V.); 9 SC Radioterapia, AO SS Antonio e Biagio e Cesare Arrigo Alessandria, 15121 Alessandria, Italy; elisabetta.garibaldi@ospedale.al.it; 10 Deptartment of Radiotherapy, Azienda Ospedaliero Universitaria S. Maria della Misericordia, 33100 Udine, Italy; alessandro.magli@asufc.sanita.fvg.it; 11 Istituto di Candiolo, Fondazione del Piemonte per l’Oncologia IRCCS, Candiolo, 10060 Torino, Italy; marco.gatti@ircc.it; 12 Programma Prostata, Prostate Cancer Program, Fondazione IRCCS Istituto Nazionale dei Tumori, 20133 Milan, Italy; 13 Deptartment of Oncology and Hemato-Oncology, Università degli Studi di Milano, 20122 Milan, Italy

**Keywords:** prostatectomy, adjuvant radiotherapy, salvage radiotherapy, urinary incontinence, ICIQ-SF, EPQ-R

## Abstract

**Simple Summary:**

The levels of urinary incontinence (UI) at adjuvant/salvage radiotherapy (ART/SRT) start strongly influence long-term UI recovery, possibly inducing clinicians to postpone radiotherapy “as much as possible” in order to maximize UI recovery, but possibly reducing radiotherapy efficacy. Our study analyzed UI recovery from prostatectomy to ART/SRT by means of the International Consultation on Incontinence Questionnaire-Short Form filled-in by patients at ART/SRT start. Three endpoints were investigated: frequency and amount of urine loss and the “subjective” patient-perceived detrimental impact on quality of life, as well as the possible influence of clinical and personality variables. The time elapsed from prostatectomy to radiotherapy start was the strongest predictor for each UI endpoint, all improving between four and eight months after prostatectomy, but without any additional long-term recovery.

**Abstract:**

Background: Baseline urinary incontinence (UI) strongly modulates UI recovery after adjuvant/salvage radiotherapy (ART/SRT), inducing clinicians to postpone it “as much as possible”, maximizing UI recovery but possibly reducing efficacy. This series aims to analyze the trend of UI recovery and its predictors at radiotherapy start. Methods: A population of 408 patients treated with ART/SRT enrolled in a cohort study (ClinicalTrials.gov #NCT02803086) aimed at developing predictive models of radiation-induced toxicities. Self-reported UI and personality traits, evaluated by means of the International Consultation on Incontinence Questionnaire-Urinary Incontinence Short Form (ICIQ-SF) and Eysenck Personality Questionnaire - Revised (EPQ-R) questionnaires, were assessed at ART/SRT start. Several endpoints based on baseline ICIQ-SF were investigated: frequency and amount of urine loss (ICIQ3 and ICIQ4, respectively), “objective” UI (ICIQ3 + 4), “subjective” UI (ICIQ5), and “TOTAL” UI (ICIQ3 +4 + 5). The relationship between each endpoint and time from prostatectomy to radiotherapy (TTRT) was investigated. The association between clinical and personality variables and each endpoint was tested by uni- and multivariable logistic regression. Results: TTRT was the strongest predictor for all endpoints (*p*-values ≤ 0.001); all scores improved between 4 and 8 months after prostatectomy, without any additional long-term recovery. Neuroticism independently predicted subjective UI, TOTAL UI, and daily frequency. Conclusions: Early UI recovery mostly depends on TTRT with no further improvement after 8 months from prostatectomy. Higher levels of neuroticism may overestimate UI.

## 1. Introduction

Adjuvant radiotherapy (ART) was believed to reduce the risk of recurrence after radical prostatectomy (RP) in patients with aggressive prostate cancer (PCa). However, three recently published non-inferiority phase III trials (RAVES, RADICALS, GETUG17), as well as the ARTISTIC meta-analysis, clearly indicated that salvage radiotherapy (SRT) at the time of recurrence may now be regarded as a preferred option in the large majority of patients [1,2,3,4].

Immediate ART could theoretically still be considered a valuable option in the presence of few pathological pejorative findings such as positive surgical margins and/or lymph-nodal metastases: according to the ARTISTIC meta-analysis, owing to the low overall event rate, the power of the patient subgroup analyses indicating no superiority of ART when compared to early SRT, and also in the subset of men with positive surgical margins, was limited [1]. Similarly, the very low number of patients with positive lymph nodes enrolled in the three trials considered in the ARTISTIC meta-analysis prevented a thorough evaluation of the effect of radiotherapy timing in this patient subset.

A strong argument favoring SRT is the common belief that postponing post-prostatectomy irradiation until a better level of urinary incontinence (UI) has been achieved may result in a reduced risk of long-term radiotherapy-induced impairment of UI recovery [5]. This is indeed a crucial issue since the key role of UI with respect to both patient’s preferences regarding the optimal management of newly diagnosed PCa [6] and long-term regret about the treatment received [7] is well recognized.

In 2016, Tendulkiar et al. elegantly highlighted an inverse relationship between pre-SRT baseline PSA and the risk of distant metastases and death following SRT, with men receiving SRT at PSA levels ranging from 0.01 to 0.20 ng/mL exhibiting better oncological outcome across all Gleason score subgroups [8], in agreement with the findings of a radiobiological model derived from a large multi-Institute database [9]. Nevertheless, even if a very early initiation of SRT may independently reduce the risk of subsequent disease progression [2,3,8,10], some reports indicate that delaying post-prostatectomy radiotherapy (even in the case of ART) could exert a protective effect on long term UI recovery after RP [11,12,13]. Although this issue is still controversial [14,15,16,17], clinicians may increasingly have to deal with the dilemma of postponing SRT despite a rising PSA until satisfactory urinary continence is achieved, or proceed to immediate SRT at the first evidence of detectable PSA values regardless of an incompletely recovered pre-radiotherapy UI. In addition, it has been generally well ascertained that rigorous studies dealing with “patient-reported” UI, which detail patient’s quality of life better than “physician-reported” UI, are lacking [18]. It is equally acknowledged that baseline pre-radiotherapy UI is a strong predictor of long-term UI after radiotherapy [13,19].

Starting from these two assumptions, we sought to analyze the trend of UI recovery from RP to post-prostatectomy radiotherapy in a large cohort (*n* = 408) of patients treated with post-prostatectomy ART or SRT.

Special emphasis was placed on the possible identification of an optimal timing at which the start of postoperative radiotherapy could be regarded as adequately safe with respect to UI recovery.

The impact of clinical factors on baseline pre-radiotherapy UI and the possible influence of patient personality on the self-assessment of UI were also investigated.

## 2. Patients and Methods

### 2.1. The IHU-WPRT TOX Study

The multi-Institutional IHU-WPRT TOX study (https://www.clinicaltrials.gov/ (accessed on 1 June 2021) identifier #NCT02803086) started in January 2014 with the goal of developing predictive models of hematological [20,21], patient-reported intestinal [22], and urinary toxicity from radiotherapy for PCa, including the prophylactic irradiation of the pelvic lymph-nodal area (whole pelvis radiotherapy; WPRT) for patients treated with ART, SRT, or with radical intent. The study was approved by the Institutional Review Board of both the coordinating Centre (San Raffaele Scientific Institute, Milan, protocol code 42/2014, date of approval 6 February 2014) and of all the participating Institutes, and is still enrolling patients [20].

A single-institute pilot study, approved by the Institutional Review Board, was previously activated in October 2012 at the Coordinating Institute (San Raffaele Scientific Institute, Milan, protocol code 33/2012, date of approval 11 October 2012) [21,22]. Both studies were conducted according to the guidelines of the Declaration of Helsinki. Informed consent was obtained from all subjects involved in the two studies.

### 2.2. The ICIQ-SF Questionnaire—Objective and Subjective UI

According to the protocol requirements, the validated Italian version of the ICIQ-SF questionnaire (International Consultation on Incontinence Questionnaire-Short Form) [23] is to be filled in by the enrolled patients at baseline, at RT mid-point and end, 3 and 6 months after radiotherapy conclusion, and thereafter every 6 months up to 5 years. The ICIQ-SF comprises six items: questions 1 and 2 (dealing with personal data) and question 6 (descriptive) do not generate a score and were not considered. Questions 3 (hereafter ICIQ3, score 0–5) and 4 (ICIQ4, score 0–6) focus on the frequency and amount of urine loss, respectively.

The sum of the two scores, ICIQ3 + ICIQ4, pertains to the “objective” (OBJ) component of UI. Question 5 (ICIQ5, score 0–10) pertains to the subjective (SUBJ) patient-perceived impairment of quality of life attributable to UI. The total score (hereafter TOTAL, ICIQ3 + ICIQ4 + ICIQ5) ranging from 0 (best) to 21 (worst), therefore comprises both an “objective” and a “subjective” component. It is rather intuitive that QoL impairment deriving from similar frequency and amount of urine loss may be perceived differently by patients, and therefore all of the three distinct aspects of radiation-induced urinary incontinence, OBJ, SUBJ, and TOTAL will be separately analyzed in the present analysis.

### 2.3. Role of Personality

Patient personality and its impact on UI were considered by means of the abbreviated 24-item version of the revised Eysenck personality questionnaire (EPQ-R) [24] filled in by patients at baseline. Four personality traits, each scored from 0 to 6, were evaluated: extraversion (sociability, impulsiveness, but also some tendency to aggressiveness), neuroticism (emotional instability, nervousness, and general anxiety), psychoticism (tough-mindedness, but also a measurement of hostility), and lie (behaviors that are either socially desirable but infrequently practiced or frequently practiced but socially undesirable).

### 2.4. Patient Population

This analysis pertains to the first 408 patients treated with ART (*n* = 179, 44%) or SRT (*n* = 229, 56%): 103 were from the pilot study [20,21] and 305 from the observational protocol [22], enrolled in 11 Italian Institutes between 2011 and 2019 and for whom a complete ICIQ-SF at baseline was available. See Table 1 for patient characteristics.

With respect to the indication of WPRT, provided that it was at the discretion of the referring radiation oncologist of every participating Institute, it was usually advised for patients with seminal vesicle invasion, Gleason score ≥ 7, pre-surgical PSA > 10 ng/mL and/or histologically positive lymph-nodes at prostatectomy, or in the case of a PSA ≥ 0.50 ng/mL in the salvage setting.

### 2.5. ICIQ Based Incontinence Endpoints

The ICIQ based endpoints were selected following, as a reference, a previous analysis on UI in the radical setting [19] and evaluating the distribution of the ICIQ scores (Table S1) in order to establish clinically relevant criteria. In summary, the following five endpoints scored at baseline were considered:

(a) daily frequency: ICIQ3 > 2
(b) amount of urine loss: ICIQ4 > 2
(c) subjective: ICIQ5 > 4
(d) objective: ICIQ3 + ICIQ4 > 5
(e) total: ICIQ3 + ICIQ4 + ICIQ5 > 8

### 2.6. Statistical Methods

#### Relationship between ICIQ Scores and Time from Prostatectomy

First, the relationship between UI at radiotherapy and the time elapsed between prostatectomy and radiotherapy (time to radiotherapy, TTRT) was investigated. The scatter plot of each ICIQ-based score versus TTRT was represented. Data were grouped into deciles with respect to the distribution along the TTRT axis: mean values and their standard errors were considered to be representative of each decile interval. Points were fitted by a sigmoid curve with initial parameters manually selected and then optimized by the *nls* function implemented in the R package *stats.* Coefficient estimation was based on an iterative process, which entails a linearization approximation leading to a least-squares problem at each step.

Subsequently, the best cut-off values for the TTRT, which best discriminate the highest risk of experiencing each of the five previously defined endpoints were identified by means of ROC curve analyses.

### 2.7. Logistic Regression Analysis and Internal Validation

The possible correlation between clinical and EPQ-R variables (see Table 2) with each endpoint tested by univariable logistic analysis.

Mean imputation was performed for EPQ-R variables when only one answer for each personality trait was missing (*n* = 23). For 46 patients, the EPQ-R questionnaire was not available owing to patient refusal or largely incomplete forms.

Variables with a *p*-value < 0.1 at univariable analyses were entered into a backward stepwise multivariable (MVA) logistic regression model. Each model was then reprocessed using only the variables retained by the MVA model with a *p*-value threshold ≤ 0.1. The multivariable analyses were subsequently repeated, excluding the personality variables.

Goodness of fit was assessed by means of the Hosmer and Lemeshow test (H&L) and the calibration of the models evaluated through the calibration plot (slope and regression coefficient R^2^). Internal validation was performed by 1000 bootstrap resampling: the Brier scores after bootstrapping were considered with and without optimism taken into account. Analyses were performed with MedCalc^®^ version 12.1.4.0 (MedCalc Software, Mariakerke Brussels, Belgium) and the R software version 3.2.4 (©The R Foundation for Statistical Computing, Vienna, Austria).

### 2.8. “Completely Dry” Patients

The predictors of the “completely-dry” condition (ICIQ3 + ICIQ4 = 0, 27% of the population) at post-prostatectomy radiotherapy start were analyzed with the same procedure followed for the identification of the predictors of the five above described UI endpoints.

## 3. Results

### 3.1. Relationship between Baseline ICIQ Scores and TTRT

**Figure 1:**
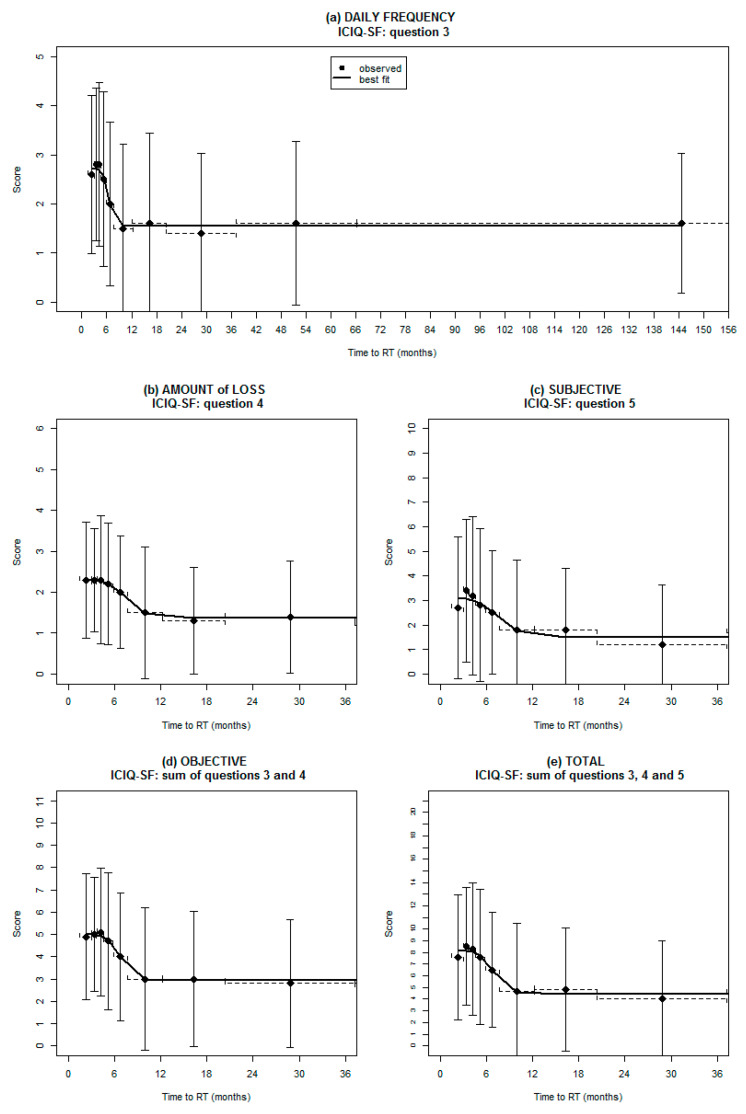
A plot of the relationship between incontinence symptoms and time elapsed between prostatectomy and post-prostatectomy radiotherapy. Incontinence was evaluated through the International Consultation on Incontinence Questionnaire Short Form (ICIQ-SF). Points represent the mean values of the ICIQ score in each decile interval; bars show the standard deviations and continuous lines and the fit between the data and a sigmoid curve. Each plot is associated with the frequency (**a**) and amount (**b**) of urine leakage, the subjective perceived quality of life (**c**), the objective (**d**), and total (**e**) estimation of urinary incontinence, respectively. The time axis for endpoint (**a**) covered the last decile to show the entire plateau (the same behavior was found for all the remaining endpoints). For the remaining endpoints, the scale was restricted to the first 3-years to render the results more easily comprehensible.

The relationships between TTRT and the different ICIQ scores are plotted in Figure 1.

All figures show an initial post-surgical plateau of the different UI aspects followed by a partial recovery and then by a new and definitive plateau. The most informative cut-offs in terms of TTRT with respect to the five previously defined UI endpoints ranged from 6.7 to 7.8 months. The coefficients of the sigmoid curves, the corresponding *p*-values, and the best cut-off values are listed in Table S2.

### 3.2. Predictors of Pre-Radiotherapy UI

The results of the univariate logistic regression are shown in Table 2. Not unexpectedly, TTRT emerged as the strongest predictor with respect to all the considered endpoints (*p*-value always ≤ 0.001). With respect to the clinical variables, the *p*-values for androgen deprivation therapy, pathologic T-stage, and pre-RP PSA were always <0.1 with the sole exception of the SUBJ endpoint, while, with respect to the role of patient personality, neuroticism always emerged as a significant factor, unlike the other three personality traits. Of note, no correlation between the type of surgery and any of the considered endpoints was observed (*p*-value always >0.2).

The resulting multivariate models, shown in Table 3, confirmed TTRT as the strongest predictor of UI, with odds ratios (ORs) ranging from 2.55 for SUBJ to 3.57 for daily frequency (ICIQ3), and resulting as the only independent predictor for the amount of urine loss (ICIQ4) and OBJ endpoints.

Interestingly, neuroticism was confirmed as an independent predictor of SUBJ and TOTAL UI as well as, quite unexpectedly, of daily frequency.

The goodness of fits were always satisfactory (H&L >0.05 and Brier score <0.216). For the models including more than one variable, the calibration showed excellent performance, with slopes ranging from 0.95 to 1.11, and with R^2^ ranging from 0.63 to 0.84. All calibration plots are shown in Figure S1. The bootstrap-based internal validation confirmed the robustness of the results: the Brier scores corrected for optimism changed by only 0.002–0.004 relative to the values of the original model.

The corresponding models obtained excluding the EPQ-R variables are shown in Table S3 and Figure S2. When disregarding the possible influence of patient personality, the independent role of age in increasing the risk of both daily frequency and OBJ (OR 1.03 and 1.04, respectively) emerged (Table S3).

### 3.3. Predictors of the “Completely-Dry” Condition

**Figure 2:**
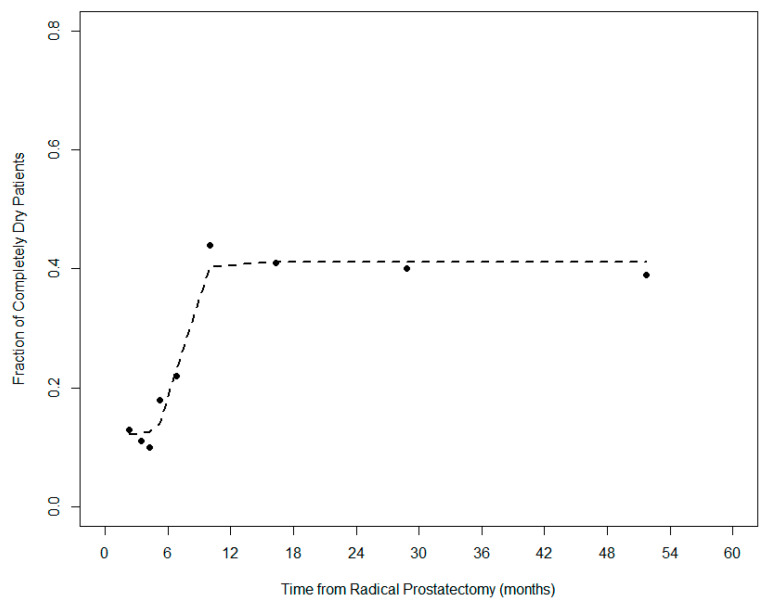
A plot of the fraction of “completely dry” patients (showing ICIQ3 + ICIQ4 = 0) against the time between prostatectomy and radiotherapy. The rate increased sharply between 4 and 10 months after surgery, reaching a plateau value of around 40% even at much longer periods.

A summary of the results of univariate and multivariate analyses are shown in Table S4 and Table 3, respectively. The two most significant predictors, confirmed by multivariate analysis, were age and TTRT, indicating a higher probability of being “completely-dry” at post-prostatectomy radiotherapy for younger (≤69 years) patients starting radiotherapy ≥7 months after surgery. The resulting two-variable model showed an excellent calibration (slope = 0.956, R^2^ = 0.837). These results were confirmed even after having excluded the role of personality (Table S3). In Figure S3 the fraction of “completely dry” patients against the time between prostatectomy and radiotherapy was shown according to patient age (≤69 years vs. >69 years). In Figure 2, the relationship between the rate of “completely-dry” patients and TTRT is plotted: the rate rapidly increased between four and ten months after surgery from about 10 to 40%, with no further improvements for longer periods after surgery.

## 4. Discussion

To the best of our knowledge, this is the first analysis of a large cohort of patients treated with post-prostatectomy radiotherapy focused on both the time-dependence of patient-reported UI recovery after radical prostatectomy and the identification of clinical and personality-related variables independently predictive of baseline pre-radiotherapy UI in its various and complex facets.

The first (and relatively unexpected) finding was that no further significant improvement of pre-radiotherapy baseline UI was recorded beyond 7–8 months after prostatectomy.

As pre-radiotherapy baseline UI was expected to be the strongest predictor of post-irradiation late UI [19,25,26], this finding heavily undermines the common propensity to postpone the beginning of radiotherapy for as long as possible in order to improve urinary outcome after ART or SRT [11]. Of note, some patients are candidates to receive ADT in combination with both ART and SRT, and for them, the postponement of irradiation is therefore of less importance. According to our findings, all the extremely multifaceted aspects of UI showed a similar relationship with TTRT, with an initial quasi-plateau up to 3–4 months from prostatectomy characterized by the highest (worst) ICIQ mean scores, followed by a rapid recovery in the subsequent 3–4 months and by a second plateau with much lower (though significantly greater than zero) ICIQ scores, but with no evidence of further recovery, even years after prostatectomy. Based on this result, the optimal “compromise” between the earliest possible postoperative irradiation and the best UI recovery falls within the range of 7–8 months after surgery, which should result in a 2.5–3.5 fold reduction of the probability of starting radiotherapy with moderate to severe UI still present. As baseline UI is expected to impact post-radiotherapy UI recovery, our results are consistent with those of van Stam and coworkers, who observed that patients starting SRT seven months or more after RP were more likely to recover urinary function after irradiation [13].

If further confirmed, these findings could lead to a dramatic reduction in the current, apparently unbridgeable, gulf between ART and early SRT.

No significant differences in terms of UI were observed among patients managed with different surgical approaches, as already noted [27,28,29]. Nevertheless, our findings should be interpreted with extreme caution, owing to the possibly heterogeneous skill levels of surgeons operating in the Institutes where the study was conducted.

Interestingly, one-fourth of the patients included in this analysis were completely dry at the start of post-prostatectomy radiotherapy, representing undeniable proof of the significant improvement in the surgical management of prostate cancer. The complete recovery from UI before post-prostatectomy radiotherapy was, as expected, more frequent in younger patients and strongly related to TTRT (>7 months). Nevertheless, the rate of the completely dry patients (which significantly increased up to 40% in patients who started radiotherapy ten months after surgery), permanently plateaued, even when several years had elapsed from surgery; clearly indicating that waiting more than ten months after prostatectomy does not further increase the likelihood of starting radiotherapy in a “completely dry” condition.

As a final remark, higher levels of neuroticism, i.e., emotional instability, nervousness, and general anxiety, were associated with a reduced capacity to cope with UI, leading to an increased perception of impaired QoL deriving from UI, but also to a possible overestimation of daily frequency (ICIQ3 score >2, OR 1.18, Table 3).

Excluding the personality-related variables, an independent role of age in increasing the risk of UI (Table S3), as already occasionally reported [13,30], has emerged. This role appeared to be even more notable when focusing on the subset of “completely dry” patients, with an OR of 0.94/year.

An important limitation of the current analysis, based on a prospective study originally aimed at investigating the predictors of acute and late radiation-induced toxicities, is the lack of any pre-surgical assessment of urinary functionality as well as the lack of information regarding perineal re-education and medications for urinary frequency after surgery.

## 5. Conclusions

Both objective and subjective UI improved notably in the first 7–8 months after RP in candidates for post-prostatectomy radiotherapy, but with no further significant recovery for longer periods after surgery.

Higher levels of neuroticism may lead to some overestimation of UI.

## Figures and Tables

**Table 1:** Summary of patient characteristics. Data are presented as counts (percentages in brackets) for categorical variables and as median values (interquartile range in bracket) for continuous variables.

Age (years)	67	(62–71)
BMI (kg/m^2^)	26	(24–28)
Time to RT (mo)	7.7	(4.1–26.4)
PSA (ng/mL)		
pre-RP	8.58	(5.83–13.98)
post-RP	0.04	(0.01–0.15)
pre-RT	0.22	(0.05–0.48)
Number of removed lymph nodes	13	(7–21)
Hypertension	178	(44%)
Smoke	69	(18%)
Diabetes	27	(7%)
ADT		
No ADT	213	(58%)
Bicalutamide monotherapy	33	(9%)
LH-RH	89	(24%)
CAB	30	(8%)
Surgical margins		
Negative	237	(58%)
Positive	171	(42%)
Surgery		
Open	233	(59%)
Robotic	120	(30%)
Laparoscopic	43	(11%)
Gleason score		
ISUP Groups 1–3	102	(27%)
ISUP Groups 4–5	274	(73%)
Stage T		
pT2	121	(30%)
pT3a	143	(35%)
pT3b and pT4	139	(34%)

BMI = body mass index; RP = radical prostatectomy; RT = radiotherapy; ADT = androgen deprivation therapy; CAB = combined androgen blockade.

**Table 2:** Odds ratios (95% CI in brackets) and *p*-values resulting from univariable logistic regression analyses: correlation with the ICIQ based endpoints. *p*-values < 0.1 are in bold.

Parameter		Daily Frequency	Amount of Urine Loss	Subjective	Objective	Total
**Age (yr)**		1.03 (1.00–1.06) **0.062**	1.00 (0.96–1.05) 0.812	1.01 (0.97–1.04) 0.697	1.03 (1.00–1.06) **0.080**	1.02 (0.99–1.05) 0.189
**BMI (kg/m^2^)**		1.05 (0.99–1.11) **0.091**	1.05 (0.97–1.13) 0.218	1.06 (0.99–1.13) 0.112	1.04 (0.98–1.10) 0.230	1.06 (0.99–1.12) **0.082**
**PSA (ng/mL)**	**pre-RP**	1.02 (1.00–1.04) **0.024**	1.01 (0.99–1.03) 0.134	1.01 (1.00–1.03) 0.104	1.01 (1.00–1.03) **0.032**	1.01 (1.00–1.03) **0.063**
	**post-RP**	0.97 (0.77–1.19) 0.775	1.14 (0.88–1.41) 0.250	1.00 (0.73–1.25) 0.981	1.00 (0.78–1.22) 0.971	0.99 (0.76–1.22) 0.922
	**pre-RT**	0.98 (0.90–1.05) 0.587	1.04 (0.96–1.11) 0.303	1.02 (0.94–1.09) 0.518	1.00 (0.93–1.07) 0.977	0.99 (0.91–1.06) 0.846
**Time to RT (mo)**						
**≥cut-off time**		Ref.	Ref.	Ref.	Ref.	Ref.
**<cut-off time**		3.30 (2.20–5.00) **<0.0001**	3.55 (1.94–6.81) **<0.0001**	2.31 (1.41–3.86) **0.001**	2.94 (1.92–4.57) **<0.0001**	3.16 (2.02–5.01) **<0.0001**
**N° removed lymph nodes**		1.01 (0.99–1.03) 0.368	1.02 (0.99–1.04) 0.113	1.01 (0.99–1.03) 0.275	1.01 (0.99–1.03) 0.549	1.01 (0.99–1.03) 0.424
**Hypertension**	**No**	Ref.	Ref.	Ref.	Ref.	Ref.
	**Yes**	1.16 (0.78–1.73) 0.460	1.00 (0.57–1.74) 1.000	1.09 (0.67–1.77) 0.722	1.16 (0.76–1.76) 0.495	0.99 (0.64–1.51) 0.945
**Smoke**	**No**	Ref.	Ref.	Ref.	Ref.	Ref.
	**Yes**	1.05 (0.62–1.77) 0.864	1.60 (0.80–3.07) 0.165	1.30 (0.69–2.37) 0.399	1.33 (0.77–2.28) 0.300	1.29 (0.73–2.22) 0.372
**Diabetes**	**No**	Ref.	Ref.	Ref.	Ref.	Ref.
	**Yes**	2.46 (1.12–5.71) **0.029**	1.36 (0.44–3.48) 0.553	0.46 (0.11–1.37) 0.219	1.51 (0.66–3.32) 0.313	1.41 (0.61–3.13) 0.407
**ADT**	**No**	Ref.	Ref.	Ref.	Ref.	Ref.
	**Yes**	1.54 (1.04–2.31) **0.033**	1.84 (1.05–3.25) **0.034**	1.31 (0.81–2.13) 0.274	1.52 (1.00–2.32) **0.052**	1.67 (1.09–2.58) **0.019**
**Surgery**	**Open**	Ref.	Ref.	Ref.	Ref.	Ref.
	**Robotic**	0.87 (0.55–1.36) 0.547	1.18 (0.63–2.17) 0.595	0.94 (0.54–1.61) 0.821	0.92 (0.57–1.48) 0.737	0.83 (0.50–1.35) 0.456
	**Laparoscopic**	1.07 (0.55–2.06) 0.836	1.44 (0.58–3.25) 0.406	0.99 (0.42–2.13) 0.988	1.27 (0.64–2.48) 0.483	1.22 (0.60–2.40) 0.566
**Surgical margins**						
	**Negative**	Ref.	Ref.	Ref.	Ref.	Ref.
	**Positive**	1.23 (0.82–1.86) 0.313	1.12 (0.64–1.99) 0.696	1.06 (0.65–1.75) 0.803	1.12 (0.73–1.72) 0.609	1.03 (0.67–1.59) 0.903
**Gleason score**						
**ISUP Groups 1–3**		Ref.	Ref.	Ref.	Ref.	Ref.
**ISUP Groups 4–5**		1.49 (0.94–2.41) **0.095**	1.67 (0.86–3.52) 0.153	115 (0.66–2.06) 0.631	1.5 (0.91–2.51) 0.117	1.34 (0.81–2.25) 0.263
**Stage T**	**pT2**	Ref.	Ref.	Ref.	Ref.	Ref.
	**pT3a**	1.50 (0.90–2.52) 0.117	1.53 (0.71–3.46) 0.287	1.00 (0.53–1.89) 1.000	2.06 (1.19–3.65) **0.011**	1.4 (0.8–2.47) 0.240
	**pT3b and pT4**	2.58 (1.56–4.34) **<0.001**	2.64 (1.29–5.76) **0.011**	1.51 (0.84–2.79) 0.175	2.44 (1.41–4.31) **0.002**	2.15 (1.25–3.75) **0.006**
**EPQ-R**						
**Extroversion**		0.97 (0.85–1.10) 0.617	0.90 (0.75–1.06) 0.202	0.96 (0.82–1.12) 0.555	0.93 (0.81–1.06) 0.270	0.90 (0.79–1.03) 0.134
**Neuroticism**		1.18 (1.04–1.35) **0.012**	1.16 (0.97–1.38) **0.098**	1.22 (1.04–1.42) **0.012**	1.15 (1.00–1.32) **0.049**	1.19 (1.04–1.37) **0.014**
**Psychoticism**		0.94 (0.77–1.14) 0.518	1.07 (0.82–1.38) 0.596	1.04 (0.82–1.30) 0.754	1.00 (0.81–1.22) 0.992	0.97 (0.78–1.18) 0.741
**Lie**		1.00 (0.85–1.19) 0.981	0.93 (0.75–1.17) 0.511	1.07 (0.87–1.33) 0.557	1.06 (0.89–1.28) 0.533	1.06 (0.89–1.28) 0.523

ICIQ3, ICIQ4, ICIQ5 = score of the International Consultation on Incontinence Modular Questionnaire Short Form for questions three, four and five respectively; BMI = body mass index; RP = radical prostatectomy; RT = radiotherapy; ADT = androgen deprivation therapy; EPQR = Eysenck Personality Questionnaire—Revised.

**Table 3:** Results of backward stepwise multivariable logistic regression analyses performed with the most significant variables (*p* < 0.1). The main performances of the models are also reported.

End-Po Int	Coeff. ± St. dev.	Odds Radio (95% CI)	*p*-Value
**(a) Daily Frequency endpoint: ICIQ3 > 2 (*n* = 152/372, 41%)**			
TTRT (cut-off: 6.7 mo)	1.272 ± 0.223	3.57 (2.31–5.56)	<0.0001
Neuroticism	0.167 ± 0.070	1.18 (1.03–1.36)	0.018
Constant:	−1.266		
H&L *p*-value = 0.693; Brier score = 0.216 (corrected for optimism: 0.219); calibration slope = 0.975; R^2^ = 0.840			
**(b) Amount of Loss endpoint: ICIQ4 > 2 (*n* = 59/408, 14%)**			
TTRT (cut-off: 7.2 mo)	1.266 ± 0.318	3.55 (1.94–6.81)	0.0001
Constant:	−2.544		
H&L *p*-value = 1.000; Brier score = 0.118 (corrected for optimism: 0.120); calibration slope = 1.000; R^2^ = 1.000			
**(c) Subjective endpoint: ICIQ5 > 4 (*n* = 75/372, 20%)**			
TTRT (cut-off: 7.1 mo)	0.935 ± 0.272	2.55 (1.51–4.39)	0.001
Neuroticism	0.190 ± 0.080	1.21 (1.03–1.42)	0.018
Constant:	−2.241		
H&L *p*-value = 0.458; Brier score = 0.152 (corrected for optimism: 0.155); calibration slope = 0.952; R^2^ = 0.794			
**(d) Objective endpoint: ICIQ3+ICIQ4 > 5 (*n* = 129/408, 32%)**			
TTRT (cut-off: 6.7 mo)	1.080 ± 0.221	2.94 (1.92–4.57)	<0.001
Constant:	−1.325		
H&L *p*-value = 1.000; Brier score = 0.203 (corrected for optimism: 0.205); calibration slope = 1.000; R^2^ = 1.000			
**(e) Total endpoint: ICIQ3+ICIQ4+ICIQ5 > 8 (*n* = 108/365, 30%)**			
TTRT (cut-off: 7.8 mo)	1.206 ± 0.246	3.34 (2.08–5.46)	<0.0001
Neuroticism	0.155 ± 0.075	1.17 (1.01–1.35)	0.039
BMI	0.064 ± 0.034	1.07 (1.00–1.14)	0.058
Constant:	−2.569		
H&L *p*-value = 0.454; Brier score = 0.188 (corrected for optimism: 0.193); calibration slope = 1.112; R^2^ = 0.633			
	Coeff. ± St. dev.	Odds radio (95% CI)	*p*-value
**Completely Dry endpoint: ICIQ3+ICIQ4 = 0 (*n* = 108/408, 26%)**			
TTRT (cut-off: 6.9 mo)	−1.429 ± 0.257	0.24 (0.14–0.40)	<0.0001
Age	−0.059 ± 0.017	0.94 (0.90–0.98)	0.001
Constant:	3.445		
H&L *p*-value = 0.185; Brier score = 0.176 (corrected for optimism: 0.178); calibration slope = 0.956; R^2^ = 0.837			

EPQ-R = Eysenck Personality Questionnaire Revised; ICIQ-SF = International Consultation on Incontinence Modular Questionnaire Short Form; TTRT = time-to-radiotherapy; H&L = Hosmer and Lemeshow test.

## Data Availability

All original data will be made available upon request.

## Supplementary Materials

The following are available online at https://www.mdpi.com/article/10.3390/cancers13133243/s1, Figure S1: Calibration plot of the final multivariate model related to each ICIQ based endpoint, Figure S2: Calibration plot of the final multivariate model related to each ICIQ based endpoint. EPQ-R variables were excluded from these analyses, Figure S3: Plot of the fraction of “completely dry” patients (showing ICIQ3 + ICIQ4 = 0) against the time between prostatectomy and radiotherapy, according to patient age (≤69 years vs. >69 years), Table S1: Median value and interquartile range of scores in the EPQ-R and in ICIQ-SF questionnaires at baseline (immediately before the beginning of the radiotherapy), Table S2: Coefficients, standard deviation, and *p*-value of the sigmoid curve fitting. The most informative cut-off, which discriminates each endpoint in terms of time-to-RT, is also reported, Table S3: Results of backward stepwise multivariable logistic regression analyses performed with the most significant variables (*p* < 0.1) excluding EPQ-R predictors. The main performances of the models are also reported, Table S4: *p*-values and odds ratios (95% CI in bracket) resulting from univariable logistic regression analyses related to the “completely dry” endpoint. *p*-values < 0.1 are in bold.
