# Supplementary material for: Predictors of Patient-Reported Incontinence at Adjuvant/Salvage Radiotherapy after Prostatectomy: Impact of Time between Surgery and Radiotherapy
Source: Cancers (Basel). 2021 Jun 29;13(13):3243. doi: 10.3390/cancers13133243 (PMC8269132; doi:10.3390/cancers13133243)
Supplement: Supplementary file 1 [file cancers-13-03243-s001.zip › cancers-1232862-supplementary.pdf]

cancers MDPI

Table S1. Median value and interquartile range of scores in the EPQ-R and in ICIQ-SF questionnaires at baseline (immediately before the beginning of the radiotherapy).

EPQR		
Psychoticism	1	(0 – 2)
Extraversion	4	(3 – 5)
Lie	5	(4 – 6)
Neuroticism	1	(0 – 3)
ICIQ-SF		
Daily Frequency	2	(0 – 4)
Amount of Loss	2	(0 – 2)
Subjective	1	(0 – 4)
Objective	4	(0 – 6)
Total	5	(0 – 10)

EPQ-R = Eysenck Personality Questionnaire Revised; ICIQ-SF = International Consultation on Incontinence Modular Questionnaire Short Form.

Table S2. Coefficients, standard deviation and *p*-value of the sigmoid curve fitting. The most informative cut-off which discriminates each endpoint in terms of time-to-RT is also reported.

ICIQ Based End-points	$ICIQscore = \frac{a}{1+e^{-b(TTRT-c)}} + d$		Most Informative Cut-off (Months)
	Coeff. ± St. dev.	*p*-value	
Daily Frequency	a = 1.158 ± 0.090	< 0.0001	6.7
	b = -1.537 ± 0.680	0.065	Sensitivity: 63.7%
	c = 6.501 ± 0.268	< 0.0001	Specificity: 65.8%
	d = 1.540 ± 0.046	< 0.0001	
Amount of Loss	a = 0.953 ± 0.126	< 0.0001	7.2
	b = -0.813 ± 0.405	0.092	Sensitivity: 74.6%
	c = 7.605 ± 0.753	< 0.0001	Specificity: 54.7%
	d = 1.376 ± 0.061	< 0.0001	
Subjective	a = 1.651 ± 0.374	< 0.0001	7.1
	b = -0.746 ± 0.585	0.249	Sensitivity: 66.3%
	c = 7.551 ± 1.262	< 0.0001	Specificity: 55.4%
	d = 1.509 ± 0.166	< 0.0001	
Objective	a = 2.083 ± 0.133	< 0.0001	6.7
	b = -1.242 ± 0.480	0.041	Sensitivity: 65.1%
	c = 6.832 ± 0.229	< 0.0001	Specificity: 62.0%
	d = 2.932 ± 0.063	< 0.0001	
Total	a = 3.694 ± 0.382	< 0.0001	7.8
	b = -1.177 ± 0.745	0.165	Sensitivity: 70.2%
	c = 6.991 ± 0.411	< 0.0001	Specificity: 58.2%
	d = 4.457 ± 0.183	< 0.0001	

TTRT = time-to-radiotherapy, i.e. months elapsed between radical prostatectomy and adjuvant or salvage RT.

Table S3. Results of backward stepwise multi-variable logistic regression analyses performed with the most significant variables ($p < 0.1$) excluding EPQ-R predictors. The main performances of the models are also reported.

	**Coeff. ± St. dev.**	**Odds Radio (95% CI)**	***p*-value**
**(a) Daily Frequency end-point: ICIQ3 > 2 (*n* = 171/408, 42%)**			
TTRT (cut-off: 6.7 mo)	1.260 ± 0.214	3.53 (2.32 – 5.36)	< 0.0001
Age	0.039 ± 0.016	1.04 (1.01 – 1.07)	0.014
Constant:	-3.551		
H&L *p*-value = 0.576; Brier score = 0.220 (Corrected for optimism: 0.223); Calibration slope = 0.968; R^2 = 0.866			
**(b) Amount of Loss end-point: ICIQ4 > 2 (*n* = 59/408, 14%)**			

	Coeff. ± St. dev.	Odds radio (95% CI)	p-value
TTRT (cut-off: 7.2 mo)	1.266 ± 0.318	3.55 (1.94 – 6.81)	0.0001
Constant:	-2.544		
H&L p-value = 1.000; Brier score = 0.118 (Corrected for optimism: 0.119); Calibration slope = 1.000; R^2 = 1.000			
**(c) Subjective end-point: ICIQ5 > 4 (*n* = 83/408, 20%)**			
TTRT (cut-off: 7.1 mo)	0.838 ± 0.256	2.31 (1.41 – 3.86)	0.001
Constant:	-1.826		
H&L p-value = 1.000; Brier score = 0.158 (Corrected for optimism: 0.159); Calibration slope = 1.000; R^2 = 1.000			
**(d) Objective end-point: ICIQ3+ICIQ4 > 5 (*n* = 129/408, 32%)**			
TTRT (cut-off: 6.7 mo)	1.139 ± 0.225	3.12 (2.02 – 4.89)	< 0.0001
Age	0.038 ± 0.017	1.04 (1.00 – 1.07)	0.027
Constant:	-3.862		
H&L p-value = 0.678; Brier score = 0.200 (Corrected for optimism: 0.203); Calibration slope = 0.985 ; R^2 = 0.799			
**(f) Total end-point: ICIQ3+ICIQ4+ICIQ5 > 8 (*n* = 121/408, 30%)**			
TTRT (cut-off: 7.8 mo)	1.150 ± 0.231	3.16 (2.02 – 5.01)	< 0.0001
Constant:	-1.507		
H&L p-value = 1.000.; Brier score = 0.195 (Corrected for optimism: 0.198); Calibration slope = 1.000; R^2 = 1.000			

	Coeff. ± St. dev.	Odds radio (95% CI)	p-value
**Completely Dry end-point: ICIQ3+ICIQ4 = 0 (*n* = 108/408, 26%)**			
TTRT (cut-off: 6.9 mo)	-1.429 ± 0.257	0.24 (0.14 – 0.40)	< 0.0001
Age	-0.059 ± 0.017	0.94 (0.90 – 0.98)	0.001
Constant:	3.445		
H&L p-value = 0.185; Brier score = 0.176 (Corrected for optimism: 0.178); Calibration slope = 0.956; R^2 = 0.837			

EPQ-R = Eysenck Personality Questionnaire Revised; ICIQ-SF = International Consultation on Incontinence Modular Questionnaire Short Form; TTRT = time-to-radiotherapy; RP = radical prostatectomy; H&L = Hosmer and Lemeshow test.

Table S4. p-values and odds ratios (95% CI in bracket) resulting from uni-variable logistic regression analyses related to the "completely dry" end-point. p-values < 0.1 are in bold.

	***p*-value**	**Odds-ratio**	**(95% CI)**
Age (yr)	**0.006**	0.95	(0.92-0.99)
BMI (kg/m^2)	0.505	0.98	(0.92-1.04)
PSA (ng/ml)			
pre-RP	**0.037**	0.98	(0.95-1.00)
post-RP	0.686	1.04	(0.83-1.28)
pre-RT	0.220	0.92	(0.76-1.01)
Time to RT (mo)			
≥ cut-off time	Ref.	Ref.	Ref.
< cut-off time	**< 0.0001**	0.27	(0.16-0.43)
N° of removed lymph nodes	0.928	1.00	(0.98-1.02)
Hypertension			
No	Ref.	Ref.	
Yes	0.773	0.94	(0.60-.46)
Smoke			
No	Ref.	Ref.	
Yes	0.997	1.00	(0.54-1.78)
Diabetes			
No	Ref.	Ref.	
Yes	0.179	0.48	(0.14-1.27)
ADT			
No	Ref.	Ref.	
Yes	0.100	0.68	(0.43-1.07)
Surgery			

Open	Ref.	Ref.	
Robotic	0.201	1.38	(0.84-2.24)
Laparoscopic	0.633	1.20	(0.56-2.43)
Gleason score			
ISUP Groups 1-3	Ref.	Ref.	
ISUP Groups 4-5	0.677	0.90	(0.54-1.50)
Stage T			
pT2	Ref.	Ref.	
pT3a	0.507	0.84	(0.50-1.41)
pT3b & pT4	**0.002**	0.41	(0.23-0.72)
EPQ-R			
Extroversion	0.440	0.95	(0.83-1.09)
Neuroticism	**0.065**	0.87	(0.74-1.01)
Psychoticism	0.414	1.09	(0.88-1.33)
Lie	0.464	1.07	(0.89-1.31)

ICIQ3, ICIQ4, ICIQ5 = score of the International Consultation on Incontinence Modular Questionnaire Short Form for questions three, four and five respectively; BMI = body mass index; RP = radical prostatectomy; RT = radiotherapy; ADT = androgen deprivation therapy; EPQR = Eysenck Personality Questionnaire – Revised.

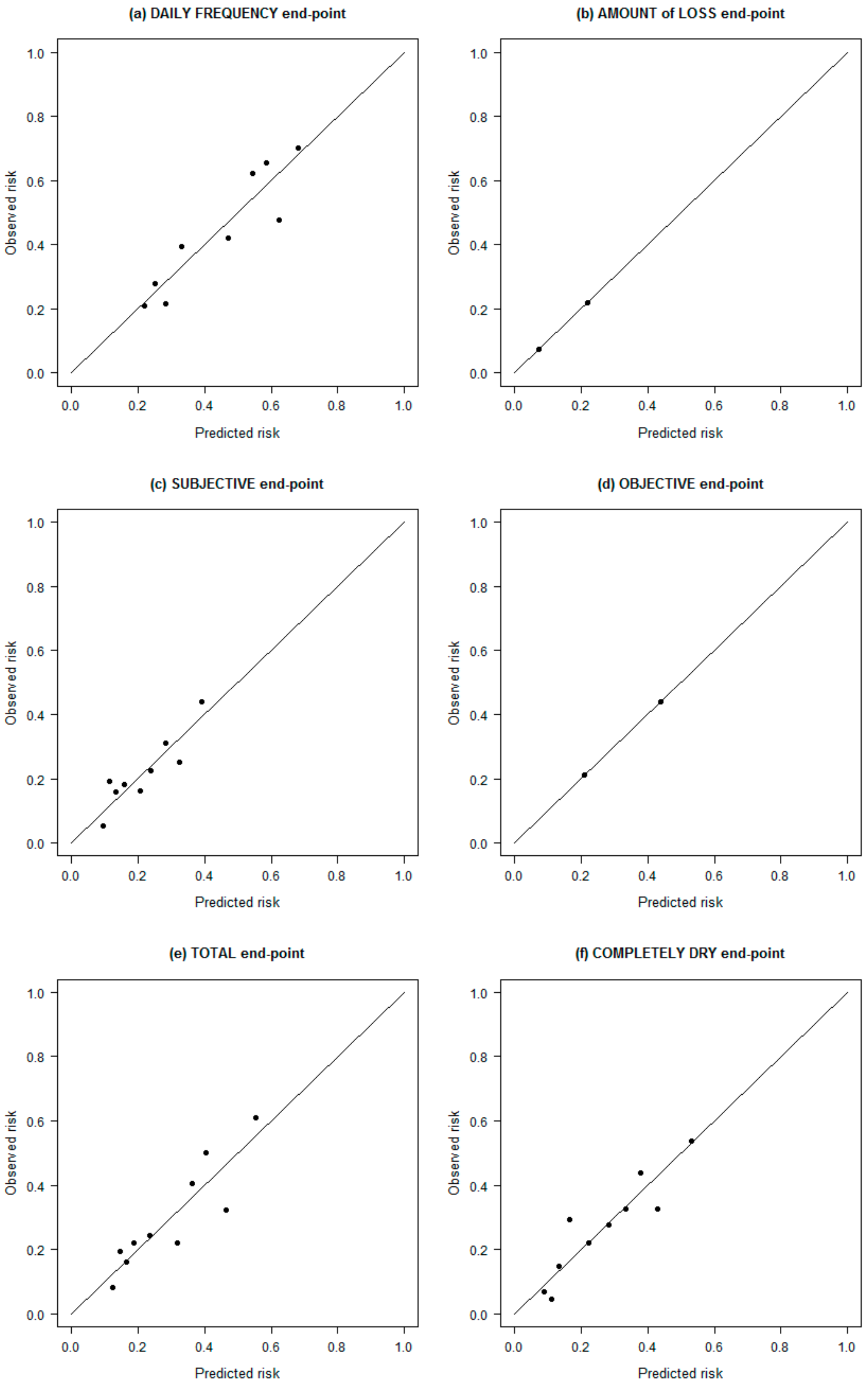

Figure S1. Calibration plot of the final multivariate model related to each ICIQ based end-point.

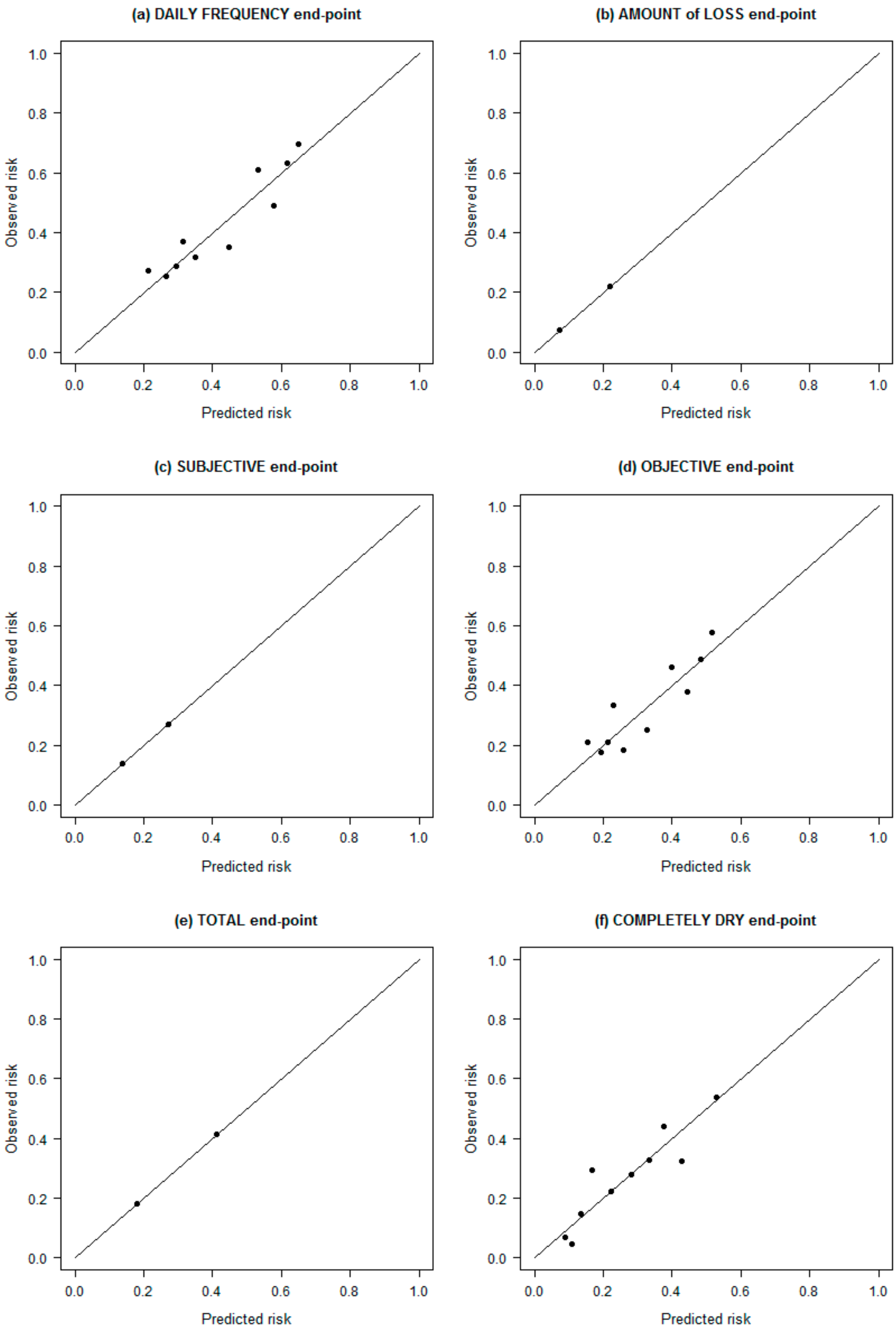

Figure S2. Calibration plot of the final multivariate model related to each ICIQ based end-point. EPQ-R variables were excluded from these analyses.

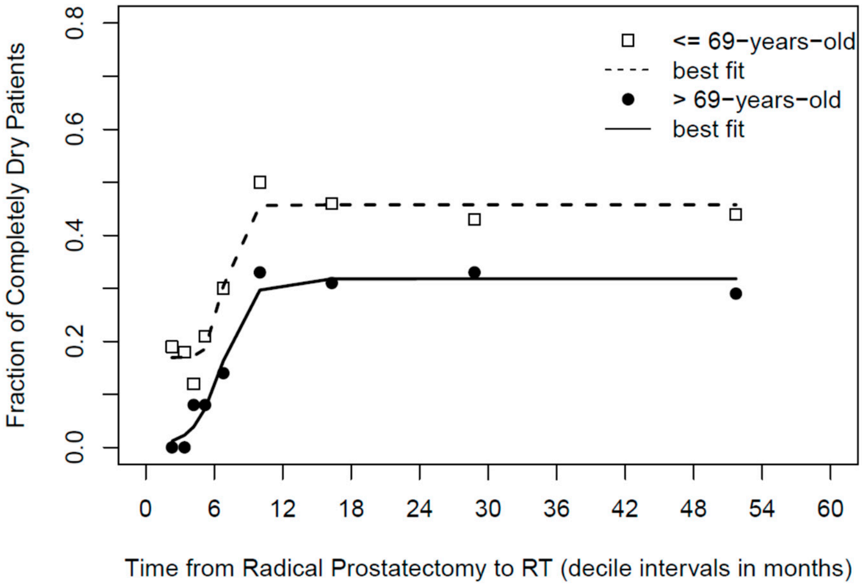

Figure S3. Plot of the fraction of "completely dry" patients (showing ICIQ3+ICIQ4=0) against the time between prostatectomy and radiotherapy, according to patient age (≤69yr vs. >69yr).
